# Whole-Genome Analysis of Three Yeast Strains Used for Production of Sherry-Like Wines Revealed Genetic Traits Specific to Flor Yeasts

**DOI:** 10.3389/fmicb.2018.00965

**Published:** 2018-05-15

**Authors:** Mikhail A. Eldarov, Alexey V. Beletsky, Tatiana N. Tanashchuk, Svetlana A. Kishkovskaya, Nikolai V. Ravin, Andrey V. Mardanov

**Affiliations:** ^1^Institute of Bioengineering, Research Center of Biotechnology of the Russian Academy of Sciences, Moscow, Russia; ^2^All-Russian National Research Institute of Viticulture and Winemaking “Magarach” of the Russian Academy of Sciences, Yalta, Russia

**Keywords:** *Saccharomyces cerevisiae*, flor yeast, sherry, genetic diversity, comparative genomics, biofilm, SNP

## Abstract

Flor yeast strains represent a specialized group of *Saccharomyces cerevisiae* yeasts used for biological wine aging. We have sequenced the genomes of three flor strains originated from different geographic regions and used for production of sherry-like wines in Russia. According to the obtained phylogeny of 118 yeast strains, flor strains form very tight cluster adjacent to the main wine clade. SNP analysis versus available genomes of wine and flor strains revealed 2,270 genetic variants in 1,337 loci specific to flor strains. Gene ontology analysis in combination with gene content evaluation revealed a complex landscape of possibly adaptive genetic changes in flor yeast, related to genes associated with cell morphology, mitotic cell cycle, ion homeostasis, DNA repair, carbohydrate metabolism, lipid metabolism, and cell wall biogenesis. Pangenomic analysis discovered the presence of several well-known “non-reference” loci of potential industrial importance. Events of gene loss included deletions of asparaginase genes, maltose utilization locus, and *FRE-FIT* locus involved in iron transport. The latter in combination with a flor-yeast-specific mutation in the Aft1 transcription factor gene is likely to be responsible for the discovered phenotype of increased iron sensitivity and improved iron uptake of analyzed strains. Expansion of the coding region of the *FLO11* flocullin gene and alteration of the balance between members of the *FLO* gene family are likely to positively affect the well-known propensity of flor strains for velum formation. Our study provides new insights in the nature of genetic variation in flor yeast strains and demonstrates that different adaptive properties of flor yeast strains could have evolved through different mechanisms of genetic variation.

## Introduction

Flor yeast strains represent a specialized group of yeasts used for centuries in various countries for biological wine aging (Alexandre, 2013; Legras et al., 2016). The physiological and biochemical properties of flor yeast strains associated with their application in specific winemaking processes are quite distinct from wine starter yeast strains and are relevant to the technological peculiarities of sherry-type wine formation (reviewed in Alexandre, 2013; Eldarov et al., 2016). One of the key prominent features of flor yeast is their capability to form a biofilm on the surface of fortified wine (Martínez et al., 1997). This ability to float is critical for flor yeast metabolic changes associated with conditions of biological wine aging and their resistance to harsh winemaking conditions. In the course of sherry wine formation, wine composition changes force flor yeasts to shift their metabolism toward oxidation of non-fermentable carbon sources leading to important changes in wine chemical composition and production of specific aromatic and flavor compounds (Peinado and Mauricio, 2009). Stressful conditions of sherry-wine formation include elevated ethanol and acetaldehyde concentration, increased oxidative damage, poor nitrogen sources, etc. Velum formation by flor yeast is generally considered as an adaptive mechanism ensuring oxygen access and resistance to harsh environmental conditions.

Taxonomic studies showed that yeast present in the velum on the surface of French and Spain sherry wines predominantly belong to *Saccharomyces cerevisiae* (Charpentier et al., 2009). They differ from wine yeast by the presence of specific 24 bp deletion or C insertion in the ITS1 region (Charpentier et al., 2009). Many flor yeasts also possess a specific deletion in the promoter of *FLO11* gene – a key cell-surface adhesin responsible for yeast cell aggregation and biofilm formation (Fidalgo et al., 2006; Voordeckers et al., 2012; Holmes et al., 2013; Legras et al., 2014). This deletion, affecting the ICR1 non-coding RNA and stimulating *FLO11* transcription, is frequent in Spanish, Italian, Hungarian, and French flor strains (Legras et al., 2014). There is a significant degree of strain variation in *FLO11*-dependent phenotypes, resulting both from variations in *FLO11* promoter and coding sequences, and *FLO11* mRNA levels (Zara et al., 2009; Barrales et al., 2012; Barua et al., 2016). Increase of the gene length is another type of *FLO11* polymorphism leading to enhancing hydrophobicity of respective yeast strains (Fidalgo et al., 2008).

These observations, however, touched only limited aspects of the specific traits of flor yeast strains, that, as other quantitative traits, are by no doubt determined by coordinated genetic and gene expression changes of numerous genes involved in cell–cell adhesion, stress resistance, nitrogen and carbon and lipid metabolism, production of aromatic compounds, etc. (Rossignol et al., 2003; Walker et al., 2014). The identification of genomic and proteomic changes specific to flor yeast was the subject of several recent studies. Microsatellite genotyping of flor yeast strains isolated in France, Italy, Spain, and Hungary have shown that most strains belong to the same genetic group (Charpentier et al., 2009). Using comparative genome hybridization, it was shown that flor strains are mostly diploid and do not have large segmental amplifications (Legras et al., 2014). Several papers report comprehensive proteome analysis of a flor yeast with regard to detecting proteins related to carbon uptake, TCA cycle, cell wall biosynthesis, mitochondrial function, and metabolism of glycerol, ethanol, and aromatic compounds (Moreno-García et al., 2015, 2017).

Due to enormous progress in next generation sequencing (NGS) methods, comparative genomics became a powerful instrument to study the origin, diversity, population structure, and natural history of *S. cerevisiae* and related yeast (Marsit and Dequin, 2015; Borneman et al., 2016;, Gallone et al., 2016). Sequencing of wine yeast genomes is the main contemporary tool to elucidate the nature of causative genetic differences underpinning the observed phenotypic variation of yeast strains, to compare the molecular genetic data with industrial characteristics of yeast strains, to study the mechanisms of yeast genome evolution under conditions of artificial selection (Bergström et al., 2014).

In a recent comparative genomic study numerous genomic loci, differentiating wine and flor yeast have been identified and phylogenetic origin of flor yeast was revealed (Coi et al., 2017). Many candidate genomic regions and regulatory networks responsible for adaptation to biological aging conditions were thus identified, providing evidence for adaptive evolution of flor yeast as a result of domestication. Importantly, genomic data confirmed that flor yeast represents a unique lineage that emerged from the wine clade through a relatively recent bottleneck event (Charpentier et al., 2009; Coi et al., 2017). Thus, a comprehensive set of statistic and genetic methods could be applied to search for genomic signatures indicating possible positive selection. Dozens of candidate genes with potentially impacting substitutions were identified, including those important for pseudohyphal growth (*IRA1, SFG1, HMS2, IME4, FLO11*, and *RGA2*) carbon metabolism (*HXT3, HXT6,7*, and *MDH2*), response to osmotic stress (*SLN1* and *SFL1*), zinc ion transport (*ZRT1*), and other processes and functions (Coi et al., 2017). The phenotypic relevance of several of identified alleles for flor yeast physiology was demonstrated using previously developed set of haploid flor strains (Coi et al., 2016).

Here, we describe the genome sequencing and comparative genomic analysis of the three *S. cerevisiae* strains used for the industrial production of sherry-type wines in Russia. We describe gene content, structural rearrangements, events of gene loss, and contribution of “non-reference” genomic material to genomic makeup of analyzed strains. By combining SNP data for our strains with those from Genowine project (BioProject PRJEB6529), we identified additional genomic regions possibly affected by positive selection. Corresponding genes with flor yeast specific alleles encode proteins involved in cell adhesion, DNA repair, carbohydrate metabolism, ion homeostasis, response to osmotic stress, lipid metabolism, cell wall biogenesis, etc. Preliminary phenotypic analysis of affected genomic loci involved in iron metabolism is provided.

## Materials and Methods

### Strains and Reference Sequences

Three flor yeast strains from the Magarach Collection of Microorganisms for Winemaking (Research Institute of Viticulture and Winemaking of the Russian Academy of Sciences) were used for genome sequencing: I-30, I-329, and I-566 (Kishkovskaia et al., 2017). The strains are available from the authors. The R64 2-1 release of the reference *S. cerevisiae* S288c genome was downloaded from Saccharomyces Genome Database (SGD)^1^ and used as reference throughout this work. The list of strains used for comparative genomic analysis is provided in Supplementary Table S1.

### DNA Isolation, Genome Sequencing, and Assembly

Cells from frozen glycerol stocks were grown on YPD plates at room temperature. Single colony was grown in 50 ml YPD at 20°C for 24 h, and cells were collected, washed in TE, and freeze-dried. Genomic DNA was prepared from freeze-dried cells with CTAB extraction method (Sreenivasaprasad, 2000) and further column purified with QIAGEN Genomic-tip 500/G kit. Final DNA concentrations were measured using Qubit Quant-iT dsDNA HS Assay kit (Thermo Fisher Scientific, United States).

The genome sequence of *S. cerevisiae* I-566 was obtained using Illumina HiSeq2500 technology. The sequencing of a TrueSeq DNA library generated 14,221,481 single-end reads (250 nt). Sequencing primers were removed using Cutadapt (Martin, 2011) and low-quality read regions were trimmed using Sickle^2^. Illumina reads were *de novo* assembled using SPAdes 3.7.1(Bankevich et al., 2012). Contigs shorter than 200 bp were discarded.

Genomes of two other strains were obtained using a combination of Illumina HiSeq2500 and PacBio RSII technologies. Strains I-30 and I-329 were sequenced using PacBio P6C4 chemistry using eight and nine SMRT cells, respectively. A total of 122,857 and 191,070 reads with an average length of 5,596 and 3,655 bp were obtained. In addition, 14,185,876 and 13,371,670 single-end reads (250 nt) were obtained upon sequencing of a TrueSeq DNA libraries using Illumina HiSeq2500. A hybrid Illumina and PacBio assembly was done using SPAdes 3.7.1 (Bankevich et al., 2012).

Protein-coding genes were predicted using Augustus 3.0.3 (Stanke and Morgenstern, 2005) trained on *S. cerevisiae* S288C dataset. Annotation of protein-coding genes was performed using BLASTP search against *S. cerevisiae* S288C proteins and a non-redundant protein sequence database. tRNA genes were predicted using tRNAscan-SE (Lowe and Chan, 2016), and rRNA genes were identified by BLASTN search against S288C rRNA genes.

For comparative genomic analysis, we also used Illumina reads previously obtained for 21 flor and wine yeast strains (Supplementary Table S1). Illumina reads were downloaded from Sequencing reads archive database and then *de novo* assembled into contigs using SPAdes 3.7.1 (Bankevich et al., 2012). Contigs shorter than 200 bp were discarded.

### Variation Identification and Genome Diversity Analysis

Illumina reads were mapped to *S. cerevisiae* strain S288C reference genome using Bowtie 2 (Langmead and Salzberg, 2012). Freebayes (Garrison and Marth, 2012) was used to find genetic variants, including SNPs, in all mapped samples.

To detect genetic variants specific for flor strains, we used a custom perl script to filter Freebayes output file. According to the filter, each sample must have a minimum 20x mapping depth in the variant position, all flor strains must support the same variant, with 90% read frequency support in each flor strain, and all wine strains can support any other allele different from the flor-specific variant, with a minimum 90% read frequency support. In total, 2,270 flor-specific genetic variants were detected using this filter (Supplementary Table S2) for the set of strains phylogenetically classified to “flor” and “wine” clades (Supplementary Table S1).

The variants were then analyzed for their non-synonymous effect on *S. cerevisiae* S288c ORFs using the Variant Annotation Integrator tool at the UCSC genome browser (Hinrichs et al., 2016). The non-synonymous to synonymous substitution rate or dN/dS ratio (Zhang et al., 2006) was calculated from the table of obtained variant calling datasets for flor yeast strain-specific SNPs and InDels.

### Phylogenetic Analysis

To analyze the phylogenetic position of selected flor strains within global yeast phylogeny, we inferred phylogenies based on multiple alignments of 16 conserved chromosomal regions suggested by Strope et al. (2015). Corresponding gene segments were extracted from the genome assemblies of strains listed in Supplementary Table S1 (except for strains WLP862 and AWRI1796) using BLAST, concatenated, and added to the collection of 218 kb sequences of 95 natural, industrial, and clinical strains downloaded from https://github.com/daskelly/yeast100genomes/. Multiple alignment was performed with MAFFT (Katoh and Standley, 2014) in fftnsi mode. Neighbor-joining tree was also constructed with MAFFT and visualized with Figtree 1.4.3 (Rambaut, 2012).

For the SNP tree, SNPs were filtered where each sample has a minimum 0.9 frequency of the major allele and a minimum 20x depth. SNPs where all major alleles for all samples are the same were excluded from tree building. Using these filters, a total of 14,069 sites were defined and concatenated into the alignment acceptable for the tree construction using a custom perl script. A maximum-likelihood tree was build using PhyML (Stamatakis, 2014). Raw sequence data and genome assemblies for flor and wine yeast strains listed in Supplementary Table S1 were used for construction of SNP-based phylogenetic tree.

### Genes of *S. cerevisiae* S288c Missing in the Analyzed Yeast Strains

Illumina sequencing reads obtained for strains I-30, I-329, and I-566 were mapped to the reference genome using Bowtie 2 and the coverage percent of each gene was calculated using Bedtools. Gene was considered as being missing when the coverage was less than 50%. In addition, we checked the absence of the “missing” genes in *de novo* assemblies by mapping contigs to the reference genome.

### Non-reference Genes Present in the Analyzed Yeast Strains

For pangenomic analysis of the presence–absence variation of key industry-related non-reference genomic segment, we used a collection of 26 sequences suggested in the recent extensive comparative genomics study of wine yeast strains (Borneman et al., 2016). Illumina sequencing reads obtained for strains I-30, I-329, and I-566 were mapped to these sequences using Bowtie 2 and the coverage percent of each gene was calculated using Bedtools.

All genes annotated in *de novo* assemblies of I-30, I-329, and I-566 genomes were compared with *S. cerevisiae* S288C genes using BLASTN search. The gene was considered as “new” in the absence of a hit with more than 70% identity over more than 80% of the gene length.

### Gene Ontology (GO) Enrichment Analysis and List Comparison

Gene sets and ORFs identified as bearing mutations or copy number alterations specific for flor yeast strains were analyzed with YeastMine tools (Balakrishnan et al., 2012) at SGD. For cases when gene ontology (GO) analysis did not show statistically significant enrichment (*p* < 0.05, Holm–Bonferroni corrected; background: SGD default) we performed GO slim term mapping and compared frequencies of the most represented terms in obtained lists versus default background.

### Other Analysis Tools

Routine sequence visualization and manipulation of nucleotide sequences was performed with Ugene (Okonechnikov et al., 2012). For drawing Venn diagrams depicting similarities and differences between various gene lists, we used the tool developed by Ghent University.^3^

#### Nucleotide Sequence Accession Number

This BioProject has been deposited in GenBank under accession number PRJNA414946. The sequences obtained in this project have been deposited in the NCBI Sequence Read Archive under the accession numbers SRR6333650, SRR6333651, and SRR6333652. The annotated genome sequences of strains I-30, I-329, and I-566 have been deposited in the GenBank database under accession numbers PTEP00000000, PTER00000000, and PTEQ00000000, respectively.

## Results

### Strains’ Origin

The “Magarach” Collection of the Microorganisms for Winemaking was started more than 60 years ago and at present harbors several hundred strains of wine-making microflora of yeast origin. Several yeast strains belonging to the group of flor yeast were either isolated from different wineries of the former Soviet Union and other countries or obtained from other collections (Kishkovskaia et al., 2017). Some strains were subjected to mutagenesis and selection for increased ethanol tolerance and velum formation properties. The biochemical, physiological, genetic, and winemaking properties of 16 flor yeast strains were re-evaluated in our recent study (Kishkovskaia et al., 2017). Three strains with superior sherry-making properties and shown to be genetically distinct according to microsatellite markers, ITS, and interdelta genotyping were subjected to *de novo* whole genome sequencing. Strains I-566 and I-30 were isolated from wineries producing sherry-like wines in Armenia and Crimea, respectively. Strain I-329 was obtained by N. F. Sayenko from a Spanish sherry winery more than 70 years ago and then was improved using selection methods in 2004. Strains I-329 and I-566 carry a 24 nt deletion in the ITS1 region found in Spanish sherry yeast strains, while in strain I-30, this region contains the C insertion characteristic of French Jura flor strains (Charpentier et al., 2009). Winemaking-relevant characteristics of these strains were reported in Kishkovskaia et al. (2017).

### Genome Sequencing, Assembly, and Annotation

All three Magarach flor yeast genomes were sequenced using Illumina NGS platform at about 200X coverage. In addition, about 60x coverage by PacBio long reads was obtained for strains I-30 and I-329. Final assemblies had total sizes in the range of 11,50–11,59 Mbp, consisting of 71–562 contigs with the N50 contig length between 58 and 511 kb (**Table 1**). As expected, the use of PacBio long reads considerably improved the assembly. Complete mitochondrial genomes were assembled as circular contigs in all three strains (Mardanov et al., 2017). On average, about 5,300 protein-coding genes and 300 tRNA genes were predicted in the nuclear genomes of strains I-30, I-329, and I-566 (**Table 1**).

**Table 1:** Statistics of sequencing, *de novo* assembly, and annotation of nuclear genomes.

Strain	I-30	I-329	I-566
Coverage by Illumina HiSeq-2500	264X	234X	219X
Number of Illumina reads (after filtration)	14,051,242	13,236,656	13,682,586
Average length of Illumina reads, nt	218	205	184
Coverage by PacBio RSII	59X	60X	–
Number of PacBio reads	122,857	191,070	–
Average length of PacBio reads, nt	5,596	3,655	–
Total contigs	155	71	562
Number of contigs larger than 500 bp	80	45	443
Contig N50, bp	470,360	511,336	58,253
Largest contig, bp	870,688	1,035,721	252,238
Total assembly length, bp	11,587,783	11,587,876	11,502,012
Predicted protein-coding genes	5,323	5,323	5,290
Predicted tRNA genes	285	288	284
Ty elements	472	474	594

### Phylogenetic Relationships of Wine and Flor Yeast Strains

Flor yeast strains from different countries are known to share unique origin based on microsatellite typing and population analysis (Legras et al., 2014). To assess the phylogenetic position of Magarach flor strains within the global yeast phylogeny, we used the large available *S. cerevisiae* phylogenetic tree constructed on the set of 16 conserved regions from 95 yeast strains (Strope et al., 2015). Corresponding sequences were extracted from genome assemblies of I-30, I-329, and I-566 strains, as well as from 20 other flor and wine strains from Genowine project and the collection of the Australian Wine Research Institute (Supplementary Table S1). One more strain from the Magarach collection, I-328 (Mardanov et al., 2018), was also included in the analysis.

**FIGURE 1:**
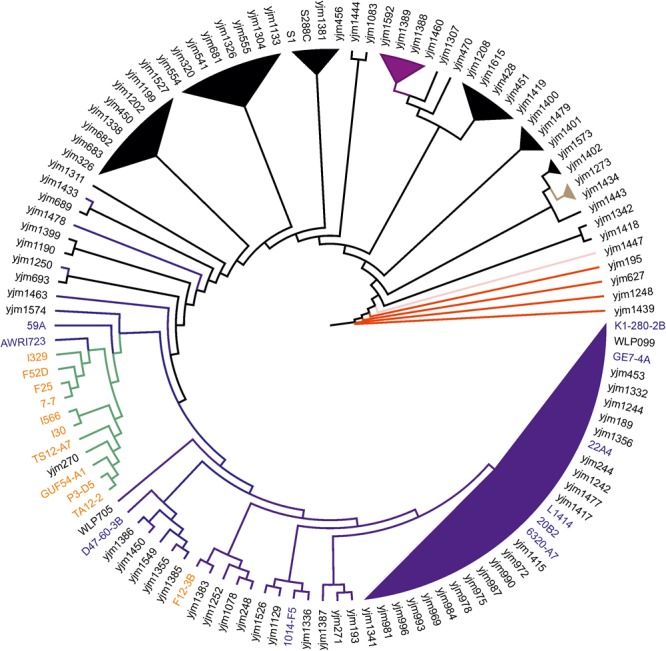
Flor yeast position on global *S. cerevisiae* phylogeny. Neighbor-joining tree of 119 yeast strains based on concatenated sequences from 16 conserved chromosomal regions. Color scheme: dark blue – Wine/European, black – mosaic, purple – Sake, red – West African, pink – Malaysian, brown – North American, and green – flor yeast clade. The names of wine and flor yeast strains from Genowine collection are outlined in blue and orange, respectively. Note that F12-3B was originally described as flor strain.

According to the obtained phylogeny of 118 yeast strains, all flor strains except F12-3B (see below) form very tight cluster adjacent to the main wine/European clade (**Figure 1**). In this cluster, strain I-329 and Spanish flor strains (FS2D, F25, 7-7) form a separate branch, and another branch comprises strains I-566 and I-30.

**FIGURE 2:**
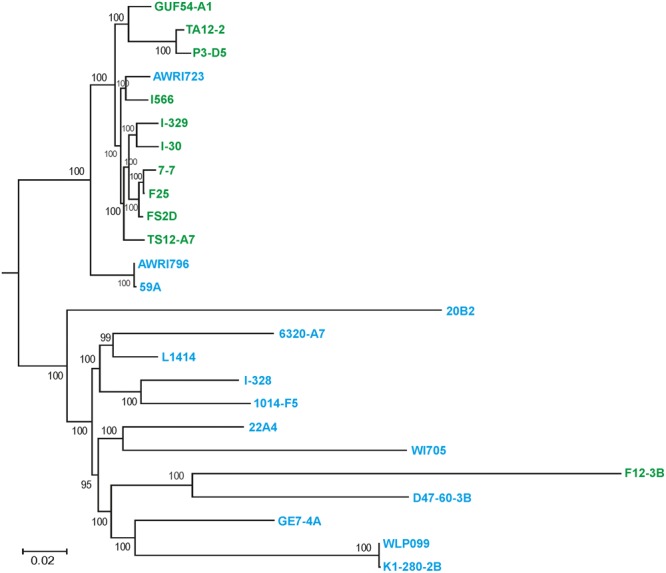
Flor and wine yeast phylogeny based on SNP analysis. A maximum-likelihood phylogenetic tree of 27 flor, wine, and lab *S. cerevisiae* strains inferred from SNP data. Numbers at nodes represent the bootstrap support values. The names of flor strains are in green, wine strains are in blue, and lab strains are in black.

These data were further refined using the whole-genome SNP-based approach similar to the one described by Coi et al. (2017). According to the obtained tree, our flor strains definitely belong to the “flor group” (**Figure 2**). They are phylogenetically related to the flor strains 7-7 (Spain), F25 (Spain), FS2D (Sardinia), TS12-A7 (Hungary), and the strain AWRI723 (Australia). The later strain was also found in the flor cluster on a phylogenetic tree constructed using the set of 16 conserved regions (**Figure 1**). On the contrary, strain F12-3B previously described as flor strain appeared to be closer to wine group on both phylogenetic trees. Strain I-328 from the Magarach collection, previously described as flor strain (Kishkovskaia et al., 2017), is phylogenetically related to the wine group.

### Gene Loss and Gain in Flor Yeast Relative to S288C

Events of gene deletion and acquisition are rather frequent in natural yeast populations and among industrial and commercial strains (Dujon, 2010; Borneman et al., 2011, 2016; Gallone et al., 2016; Marsit et al., 2017). The redundant nature of yeast genome suggests that many genes can be lost without dramatic effects on strain viability and fitness (Dean et al., 2008; DeLuna et al., 2008), but the real evolutionary implications are unclear (Sliwa and Korona, 2005). On the other hand, there are many well-documented events of gene acquisition by wine yeast species through horizontal gene transfer or introgression from other yeast or bacterial species (Galeote et al., 2010; Bergström et al., 2014). The transferred segments encode functions with a clear impact on wine fermentation such as stress resistance and improved utilization of carbon and nitrogen sources, justifying important role of this type of diversification in yeast evolution (Marsit and Dequin, 2015; Marsit et al., 2015).

The degree of gene loss in the three Magarach flor strains as determined using mapping of reads on the genome of the reference strain S288C, as well as by analysis of *de novo* assemblies, appeared to be rather low. A total of 92 genes present in strain S288C were missing in all three sequenced strains (Supplementary Table S3). No genes absent in only one or two strains were identified. These lost genes predominantly encoded either Ty transposon proteins (65), or putative proteins with unknown functions (17). The effects of the loss of 10 genes with known functions may be significant. They are located in three genomic loci.

Thus, we observed extended deletions of genes responsible for iron uptake at the subtelomeric region of chromosome XV and nearly located DNA photolyase PHR1, the asparaginase genes near rDNA array on chromosome XII, and *MAL* genes (transcriptional factor *MAL13* and maltose transporter *MAL11*) on chromosome VII. These deletions may obviously affect carbon metabolism, amino acid metabolism, and iron homeostasis.

Comparative genomic analysis of numerous wild, commercial, industrial, and clinical isolates of *S. cerevisiae* has revealed extended regions of genetic material, scattered across distinct chromosomal regions, apparently absent from the reference S288c genome (Novo et al., 2009; Dunn et al., 2012; Song et al., 2015; Borneman et al., 2016; McIlwain et al., 2016). Many of these strain-specific loci encode functions beneficial for particular industry-related traits. Well-known examples of clustered loci of industrial importance are the RTM1 cluster, important for membrane phospholipid homeostasis at high ethanol concentrations, the “wine circle” (Borneman et al., 2011), or region B, regions A and C (Novo et al., 2009) identified in wine strain EC1118, the heat-resistant toxin *KHR1*(Goto et al., 1990), the *MPR1* gene encoding L-azetidine-2-carboxylic acid acetyl-transferase conferring ethanol and cold resistance, and oxidative stress tolerance (Takagi et al., 2000). A useful compendium of these non-reference sequences was developed by Borneman et al. (2016) and we used this resource to identify non-reference sequences in our three flor yeast genomes (Supplementary Table S3).

The nuclear genomes of Magarach strains contained about 108–126 kb absent in the reference genome. All three strains lacked the so-called region A previously identified in EC1118 genome. Region B was found only in strain I-30 where it comprises five genes: transcription factor, 5-oxoprolinase, nicotinic acid transporter, flocullin-like protein, and a hypothetical protein. Region C encodes, among other genes, *FOT* oligopeptide transporters beneficial for utilization of “non-conventional” nitrogen sources. Many flor yeast strains contain this region, but region C is absent from the three our strains. Not surprisingly, the three analyzed genomes also lacked the RTM-cluster, which is known to be advantageous for beer and bioethanol strains, grown on molasses.

Potentially important for flor yeast physiology and metabolism is the presence in all three genomes of the *MPR1* gene and two other regions found in wine yeast strains (Argueso et al., 2009; Akao et al., 2011). The 5 kb segment encoding the ortholog of GPI-anchored cell-wall protein AWA1 from sake strain may positively affect surface adhesion of flor yeast cells (Shimoi et al., 2002). All three Magarach strains contained AWA1-like genes most similar to ones from wine strains YJM1341 and YJM1415. The 19 kb cluster from bioethanol strain JAY291 is known to encode a paralog of the HXT4 high-affinity glucose transporter and alpha-glucosidase MAL32, both advantageous under conditions of sugar limitation (Akao et al., 2011). These two genes are present in each of Magarach strains. In contrast to these full-length clusters, other sequences listed in Supplementary Table S3 are either missing or are represented by significantly truncated fragments. The potential role of KHR1 toxin (present in I-30 and I-566), the EC1118 1M36 cluster harboring one hypothetical protein gene (present in all three strains), and the endogenous 2 mcm plasmid (present in I-30 and I-566) is unclear.

The search for non-reference genes in *de novo* assemblies revealed one to three new genes in each strain in addition to genes located in above-mentioned regions (Supplementary Table S3). All of them encode hypothetical proteins with unknown functions. Interestingly, all three strains contained a gene which predicted product is identical to 246-aa protein R103_P20001 from *S. cerevisiae* R103. Highly similar genes were present in several other wine yeast strains (JAY291, FostersB, YJM789, FostersO, Lalvin QA23, VIN7, and VL3).

### Flor-Yeast-Specific Sequence Variations

Using variant calling, we have identified two types of variations – SNP and InDel in three Magarach flor yeast genomes, accounting in each case to more than 45,000 variable site relative to the reference S288C genome (**Table 2**). In order to narrow down this set and to find flor yeast specific mutations (FYSMs), we have compared obtained SNP sites to draft genomes of wine and flor yeast strains listed in Supplementary Table S1 and phylogenetically assigned to “wine” and “flor” clades as described in **Figure 2**. In total, we found 2,270 high-quality biallelic flor yeast specific SNV (both SNP and InDels) in 1,337 genomic loci (Supplementary Table S2) and subjected this set to different types of analyses. First, we analyzed the distribution of variable sites across the chromosomes and found significant SNV enrichment in some “hot spots,” including subtelomeric regions of several chromosomes in accordance with well-known view of these structures as “hotbeds” of genome variation in yeast (Supplementary Figure S1). Using SNPeff, we classified mutations functionally in different subcategories (**Table 2**). These new gene sets, in particular, genes with missense mutations and with mutations in promoter regions, were subjected to GO enrichment analysis to identify GO terms that are under- or over-represented compared to reference genome.

**Table 2:** SNP categories in flor strains.

Total SNP and InDels	Number
I-30	46,756
I-329	47,438
I-566	45,656
Flor yeast specific variants	2,270
Missense	982
Synonymous	583
Frameshift	8
Upstream	549
Downstream	121
Intron	4
Intergenic	8
Stop and splice	15

The ratio between missense and synonymous mutations in coding regions was high (dN/dS = 1.68), and thus we first looked for GO terms enriched in the set of genes with missense mutation in coding regions likely to be under positive selection. The GO analysis of obtained list of 670 unique genes revealed significant alterations in “cell component,” “biological process,” and “molecular function” categories relative to the reference genome (Supplementary Table S4). In particular, in “cell component” category such terms as “intracellular membrane bound organelle” and “protein complex” were enriched. In “molecular function” category, various terms such as “ATP binding” and “ATP ase activity” were enriched. In “biological process” category, we found enrichment for the following terms: “regulation of cellular process,” “response to stimulus,” “cellular component organization,” “developmental process,” “aromatic compound biosynthetic process,” and others (Supplementary Table S4). This analysis points to importance of process related to integrity of intracellular organelles, ion, and protein homeostasis for flor yeast specific physiological and biochemical features. Notably, in this list, we found 20 genes for stress-responsive transcription factors involved in reprogramming of non-fermentative metabolism, *ACC1, CAT8, LN3, ERT1, GCN4, GSY2, HAP1, LST8, MSN4, NTH1, PFK2, PHO85, PSK1, RIM15, SUT1, TCO89, TOR2, TPK2, TPK3*, and *YAK1* (Soontorngun, 2017).

In order to select ORFs likely to be under stronger positive selection, we have further divided the set of ORF with dN/dS > 1 according to the number of sites per gene. We have ranged the genes with missense mutations according to the number of SNP per gene and those with two or more missense SNP were considered as “highly polymorphic.” For this group of 106 genes (Supplementary Table S5), we performed GO slim mapping and detected prevalence for GO slim terms in all three categories. In the “biological process” category, genes involved in “response to chemical,” “transcription from RNA polymerase II promoter,” “ion transport,” “mitotic cell cycle,” “signaling,” “cellular response to DNA damage stimulus,” “transmembrane transport,” “carbohydrate metabolic process,” “DNA repair,” and others were over-represented (Supplementary Table S5). In the “molecular function” group, the following GO terms were enriched: “hydrolase activity,” “transferase activity,” “ATPase activity,” “transmembrane transporter activity,” “DNA binding,” “enzyme regulator activity,” “helicase activity,” etc. Such GO terms as “cellular bud,” “plasma membrane,” “site of polarized growth,” and others were prevalent in “cell component category.”

The small group of 25 genes with “deleterious mutations” (stop-codon lost or gained, frameshift, and altered splicing site) included proteins involved in transcription regulation and signaling, and unknown genes with unclear role for flor yeast specific adaptation (Supplementary Table S5).

Mutation in the upstream and downstream regions may positively or negatively affect gene expression. We focused on upstream mutations and selected a group of 106 genes with two or more SNPs in promoter regions and performed GO enrichment analysis. We found enrichment for terms related to cellular ion homeostasis, reflecting possible positive selection (Supplementary Table S6). Pathways’ enrichment analysis detected enrichment of gene related to acetoin biosynthesis, pentose phosphate pathway, and amino acid catabolism, all possibly related to flor yeast specific biochemical features.

The group of 25 genes with three or more SNVs in the promoter regions (Supplementary Table S7) included those related to carbon metabolism (*PDC1* and *TKL1*) and utilization of unconventional nitrogen sources (*SRY1*), aquaporin *AQY2*, and several proteins that may affect metal ion transport (ferric reductase *FRE6* and zinc transporter *YKE4*), RNA processing (*YRA1* and *MTR2*), and BET3 component of the transport protein particle. Changes in regulation of genes relevant to mitochondrial function (*SDH6, SMF1, HMX1*, and *FRE6*) may be important for flor yeast under conditions of oxidative metabolism (Supplementary Table S7).

Finally, we ranged all polymorphic genes by total number of SNP per gene (upstream, downstream, synonymous, and missense) to identify those that are most polymorphic and selected among them those with dN/dS > 1. This selection yielded a rather interesting group of 39 extremely polymorphic genes (five or more sites per gene) with functions possibly directly related to flor yeast fitness (Supplementary Table S8). Besides already identified genes with upstream mutations, we found several genes with functions related to flor yeast morphology, in particular septin ring formation (*RGA2, VHS2*, and *YCK2*) and intracellular trafficking (*VPS13, COS9*, and *SEC24*), that may contribute directly or indirectly to enhanced ability of flor yeast for biofilm formation. Modification of *DNA2* gene involved in DNA replication, double-stranded break repair, and telomere maintenance may enhance the resistance of flor yeast to mutagenic action of high ethanol and acetaldehyde concentrations. Several genes encode proteins with unknown functions and their significance for flor yeast specific properties remains to be elucidated.

### Structural Variations in Flocullins

The key role of *FLO11* in determining the ability of flor yeast for biofilm formation is well established (Fidalgo et al., 2006; Ishigami et al., 2006; Zara et al., 2009). The two sequenced strains, I-30 and I-329, carry a characteristic *FLO11* promoter deletion, known to positively affect *FLO11* transcription (Fidalgo et al., 2006). The coding regions of *FLO11* on our strains were extended due to accumulation of tandem repeats in the central domain (Supplementary Figure S2) that was shown to yield more hydrophobic Flo11p variant and increase the ability of yeast cells to float (Fidalgo et al., 2006).

The opposite trends were observed for three other adhesin genes, *FLO1, FLO5*, and *FLO9*. Full-size genes for the largest flocullin Flo1p (1537 a.a. long in strain S288C) were not found in all three flor strains; only genes able to encode 390 a.a. long protein were present. On the contrary, nearly full size *FLO5* genes were found in all Magarach strains. *FLO9* genes were also found, but the number of tandem repeats in the central domain was reduced relative to the reference gene. This balance change between the two groups of Flo proteins in flor yeast strains indicates a possible positive selection in favor of increased *FLO11* expression leading to improved velum formation.

### Phenotypic Assessment of Variations in Iron Uptake Genes

**FIGURE 3:**
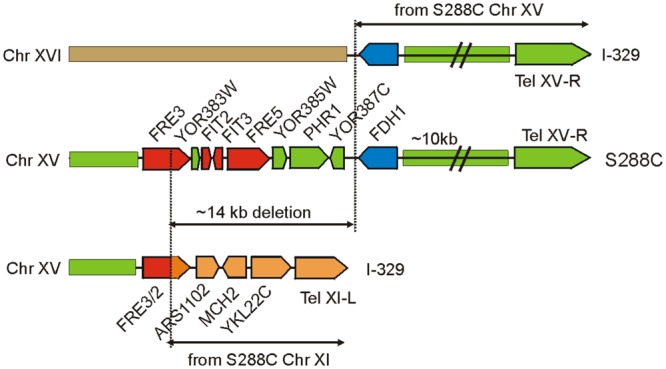
Genome rearrangements resulting in the loss of *FRE/FIT* cluster in strain I-329. *FRE/FIT* genes are shown in red, *FDH1* in blue, and other genes and genome regions are colored according to their origin from chromosomes XV, XI, and XVI.

The three sequenced Magarach flor strains possess two structural variations with a potential strong impact of iron uptake and homeostasis – the 14 kb deletion in the right subtelomeric region of chromosome XV (**Figure 3**) and a flor-yeast-specific deleterious mutation in the gene encoding Aft1 transcription factor, leading to stop-codon insertion at position 648, eliminating 42 C-terminal amino acid residues (Supplementary Figure S3).

Mapping of contigs obtained for Magarach strains to the reference genome revealed that this *FRE/FIT* deletion likely resulted from recombination between subtelomeric regions of chromosomes XV and XI (**Figure 3**). The left subtelomeric region of chromosome XI contained gene *FRE2* exhibiting high sequence similarity to *FRE3* in the *FRE/FIT* cluster on chromosome XV; recombination between these sequences produced “hybrid” *FRE2/FRE3* gene followed by genes, initially located between *FRE2* and the left telomere of chromosome XI. The 14-kbp *FRE/FIT* region appeared to be lost, while telomere-proximal region with *FDH1* gene was translocated to the chromosome XVI (**Figure 3**).

The *FRE* and *FIT* proteins are known to cooperate in iron uptake (Outten and Albetel, 2013). Fit2p and Fit3p are GPI-anchored cell-wall mannoproteins facilitating iron uptake through increasing the amount of iron associated with the cell wall and periplasm (Protchenko et al., 2001). Fre2p and Fre5p are plasma membrane reductases that facilitate uptake of siderophore-bound iron. Aft1 upregulates expression of iron uptake genes when iron is scarce and in combination with Yap5 transcription factor is essential to maintain iron homeostasis in yeast (Martínez-Pastor et al., 2017). The Q648X mutation removes C-terminal region with potential sumoylation and CK2 phosphorylation sites, leaving intact the Q-rich domain potentially involved in transcriptional activation (Supplementary Figure S3). The combination of these strong structural variations was found in other flor strains and this prompted us to directly assess its phenotypic effects through comparison of flor and lab yeast strains.

Iron is vital for aerobic flor yeast metabolism under conditions of biological wine aging, but excess iron may be detrimental due to accumulation of toxic reactive oxygen species, damaging cellular macromolecules (Bresgen and Eckl, 2015). There is a significant variation in iron uptake capabilities in natural yeast isolates leading to separation of “iron-sensitive” or “iron-resistant” groups depending on strain response to excess iron in the medium (Martínez-Garay et al., 2016). To assess the net effect of indicated structural variations on iron homeostasis and uptake of flor yeast strains, we performed growth assays similar to those described before (Martínez-Garay et al., 2016).

All three flor strains were more sensitive to excess iron in the medium compared to lab strain (Supplementary Figure S4). Growth on solid medium was inhibited at ferric iron concentration above 3 mM; in liquid medium, the retardation of cell division was observed if concentration of ferrous iron was above 1 mM and became more pronounced at 4 mM (Supplementary Figure S4). In accordance with this iron-sensitive phenotype, flor yeast strains displayed increased coloration on the plates with 2 mM ferric iron and 1% methylene blue indicating more oxidized cellular redox state in the presence of iron (Supplementary Figure S4).

Increased iron sensitivity and iron-dependent methylene blue oxidation are considered to be indicative of improved iron uptake (Martínez-Garay et al., 2016), which prompted us to propose that flor yeast strains are more proficient in iron uptake. This assumption was tested in iron accumulation assays for I-329 strain and control BY4743 strain grown at different conditions. The intracellular iron content in the iron-sensitive strain I-329 was higher under both low-iron (0.1 mM) and high-iron (4 mM) conditions, indicating its iron uptake proficiency (Supplementary Figure S4). Since no other genetic alterations in known iron uptake and homeostasis system were detected in three sequenced strains, we attribute this property to combined effect of *AFT1* mutation and *FRE/FIT* cluster deletion.

## Discussion

Flor yeast strains are highly specialized microbial agents used for production of biological aged wines through sophisticated winemaking process (Alexandre, 2013). The important properties of flor yeast, such as high tolerance to harsh environment conditions, capability for velum formation and production of specific flavor compounds are likely to have evolved through centuries of “unconscious” human selection and domestication (Legras et al., 2007, 2014). Understanding the nature of the genetic variations specifying the particular phenotypic properties of flor yeast is of major importance for the study of molecular mechanisms of yeast adaptation to industrial processes and specific ecological niches and identification of flor yeast specific genes and alleles.

Our comparative genomic approaches have revealed complex landscape of genetic variation in three newly sequenced flor strains represented by SNPs, InDels, events of gene loss and gain. Subsequent GO analysis uncovered differential contribution of different forms of genetic variation to the build-up of the flor yeast genomes. The polymorphism in the genes involved in yeast morphology, carbohydrate metabolism, ion homeostasis, response to osmotic stress, lipid metabolism, DNA repair, cell wall biogenesis, etc., in sherry strains is mainly due to SNP/InDel accumulation. On the other hand, the genes for FLO adhesins were the subject of significant structural variation that could explain the increased biofilm-formation capacity of flor yeast.

It is necessary to note the difference of our results from the results of the recent study of genomic signatures of flor yeast adaptation reported by Genowine researchers (Coi et al., 2017), although the set of strains and assemblies essentially overlapped. Our criteria for selection of flor-yeast-specific mutations were different both in terms of dataset analysis methods and the selection of affected regions. For instance, we have included mutations in regulatory regions in the set of compared SNPs. Such mutations, as recently was shown, may affect gene expression both positively or negatively not only by affecting transcription binding sites and their spacing in promoters, but also via DNA “zip codes” responsible for interaction between promoters and nuclear memory (Brickner et al., 2015) and mRNA stability sites (Shalem et al., 2015).

Superposition of our set of 670 genes with FYSM and the dN/dS ratio > 1 with the FYSM genes likely to be under positive selection identified in Genowine study showed an overlap of 89 protein-coding genes (Supplementary Table S9). This list is enriched for proteins located at the cell periphery (23 proteins), involves several genes implicated before in regulation of ethanol tolerance (*YDR274, FTR1, CCS1*, and *BRE5*), signaling (*IRA1* and *TCO89*), DNA repair (*DNA2* and *DDC1*), and transporters (*PMA1, TPO5*, and *QDR2*).

Irrespective of the differences in algorithms and approaches applied to select for FYSM genes, this comparison shows the difference in attestation of analyzed strains to flor or wine groups. For instance, the F12-3B strain originally classified as “flor yeast strain” (BioSample: SAMEA2612327) according to SNP-based and 16 conserved regions-based phylogenetic trees belongs to the “wine” clade, while the strain AWRI1723 (BioSample: SAMN 04286124) belongs to the “flor” clade. Wine strains 59A and AWRI 1796 are also phylogenetically closer to the flor group (**Figure 2**). Of course, strains phylogenetically related to wine group may perform well in biological aging due to some specific set of mutations. It is also possible that some strains originally described as “wine” but phylogenetically related to the flor clade could perform wine aging as well. Obviously, more extensive comparative genomic and post-genomic analysis of flor yeast strains is required to clarify these issues.

Only a limited number of gene acquisition and loss events were observed in three Magarach flor strains. Only two genes, missing in the reference strain S288c, were found in all three studied flor strains. The first is the *MPR1* gene coding for *N*-acetyltransferase that is involved in oxidative stress tolerance via proline metabolism (Nishimura et al., 2010). Its presence is apparently beneficial for flor strains thriving under aerobic conditions. The second gene encodes a protein with unknown function. Both genes are not unique for flor strains and were found in a number of wine yeasts. The gene loss events are mostly related to genes encoding transposon-related and hypothetical proteins, but deletions of three larger genomic loci were detected as well. Deletions of the *MAL1* locus located in the subtelomeric region of chromosome VII are rather often event in natural population and may impose no obvious phenotypic effect since five nearly identical *MAL* loci have been identified in *S. cerevisiae* (Charron et al., 1989; Naumov et al., 1994). Deletion of the asparaginase gene cluster is also quite often and is not expected to be clearly related to conditions of biological wine aging. The third deletion, targeting the *FRE-FIT* cluster, could be more important.

We took an advantage of the two potentially strongly impacting FYS-genetic variation that could be directly assessed through comparison of wild type flor and lab strains, the deletion of *FRE-FIT* cluster and mutation in *AFT1* transcription factor. Our phenotypic analysis has shown that analyzed flor strains are more sensitive to iron toxicity that is likely to be related to their increased capacity for iron uptake. This assumption was proved in our iron accumulation assays.

The adaptive significance of this trait of course requires additional evaluation. Since *FRE-FIT* genes are dispensable for iron uptake in the absence of siderophore-bound iron (Protchenko et al., 2001), their deletion may be neutral for flor yeasts growing in sterilized wine materials in course of sherry wine making. However, it is also possible that such deletion in combination with flor-yeast-specific Aft1 allele is advantageous to improve iron uptake from wine materials with low iron content.

Aft1 is a known positive activator of the iron regulon, that besides *FRE1-4* metalloreductase genes and *FIT1-3* iron siderophore transporters includes genes involved in cell-surface high-affinity iron acquisition (FET3/FTR1 system), multiple genes for proteins involved in iron recycling, intracellular transport, post-transcriptional regulation, etc. (Martínez-Pastor et al., 2017). One may expect that elimination of the *FRE-FIT* genes in flor yeast strains is compensated by activation of FET3/FTR1 system and alteration in the iron levels between cytosol, vacuoles, and mitochondria. Thus, Aft1 targets are attractive candidates for more detailed gene expression analysis in flor yeast strains under a variety of conditions and are in focus of our current investigation.

The metal content in wines is of great interest due to influence on wine technology and is determined largely by geographic origin (Galani-Nikolakaki et al., 2002). It is known that in Jerez wines, the iron content is below 0.05 mM (Paneque et al., 2009). This may be important to preserve typicality of at least some varieties of sherry wines. It is known, for instance, that Fino sherry wines undergo browning at iron concentration above 0.05 mM (Benìtez et al., 2002). The influence of FIT genes deletion on flor yeast cell wall properties should also be evaluated. Individual and combined allele replacements, iron toxicity, biofilm formation, and other assays may be required for this type of research.

We suppose that the results of our analysis, sequence data, and *de novo* assemblies will help to infer the evolutionary history and the adaptive evolution of flor yeasts. They can also be useful for functional analysis of flor yeast, for instance, through application of modern synthetic biology and genome editing tools (Jagtap et al., 2017), recently developed set of haploid flor strains (Coi et al., 2016) to aid in development of novel flor yeast with improved properties.

## Author Contributions

AM, ME, and NR designed the research project and wrote the paper. SK, TT, AB, and AM performed the research. AB, AM, NR, and ME analyzed the data. All authors read and approved the manuscript.

## Conflict of Interest Statement

The authors declare that the research was conducted in the absence of any commercial or financial relationships that could be construed as a potential conflict of interest.

## Footnotes

1 https://downloads.yeastgenome.org/sequence/S288C_reference/

2 https://github.com/najoshi/sickle

3 http://bioinformatics.psb.ugent.be/webtools/Venn/
