# Supplementary material for: Whole-Genome Analysis of Three Yeast Strains Used for Production of Sherry-Like Wines Revealed Genetic Traits Specific to Flor Yeasts
Source: Front Microbiol. 2018 May 15;9:965. doi: 10.3389/fmicb.2018.00965 (PMC5962777; doi:10.3389/fmicb.2018.00965)
Supplement: Supplementary file 10 [file Image_1.PDF]

Figure S1. Distribution of flor yeast strain specific SNV along *S. cerevisiae* S288C chromosomes.
Numbers of SNVs detected in 2000 nt window are shown.

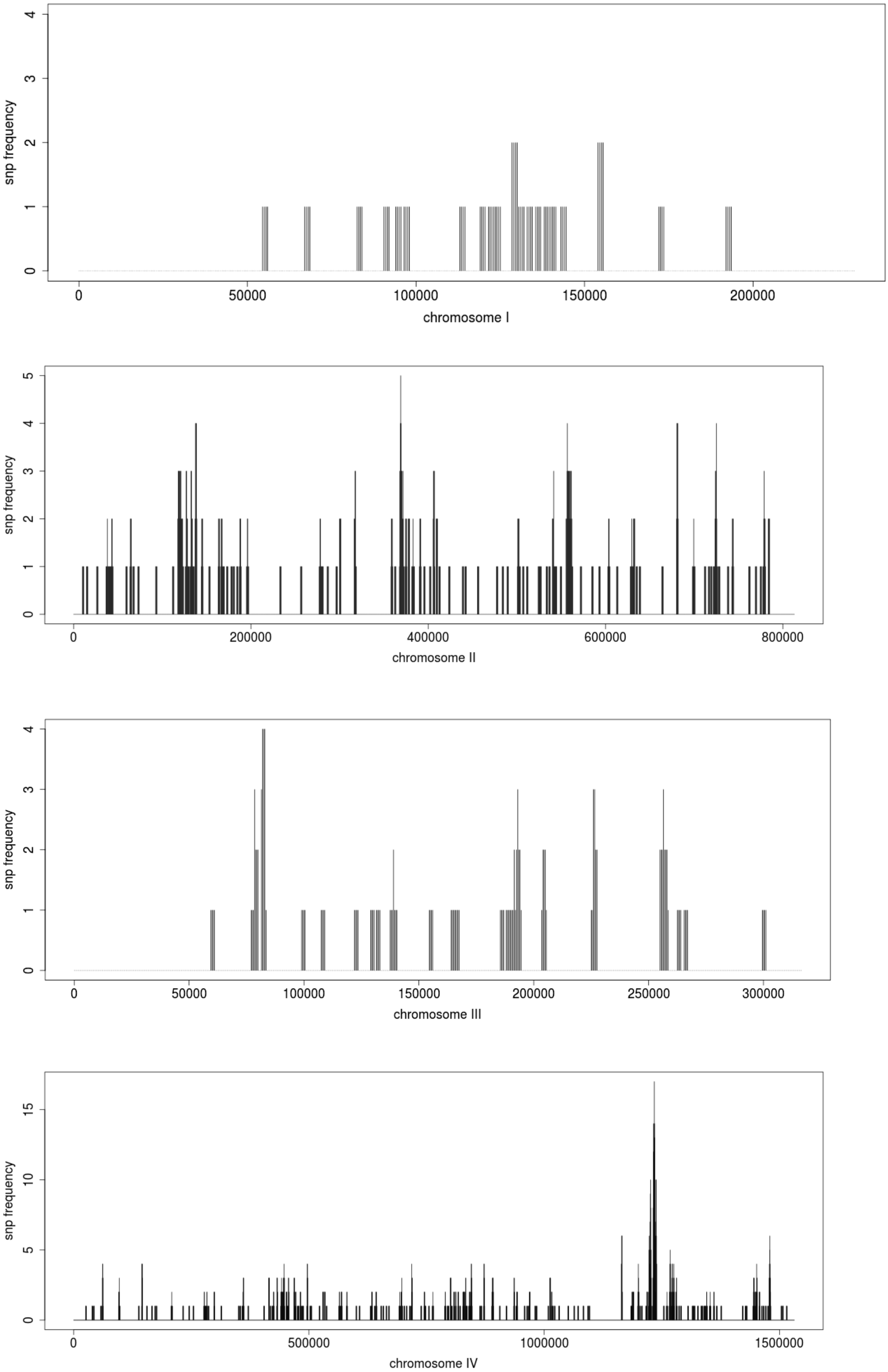

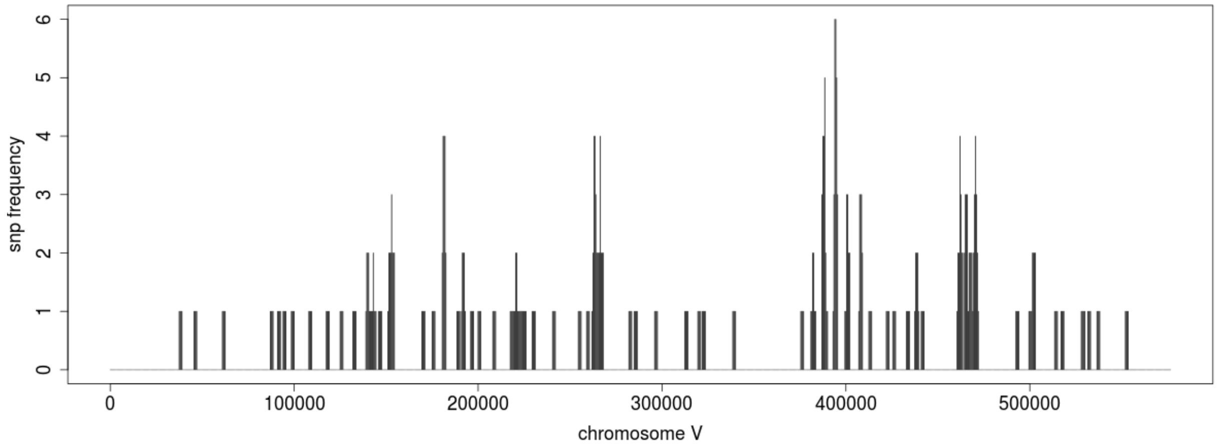
snp frequency
chromosome V
0
1
2
3
4
5
6
0
100000
200000
300000
400000
500000

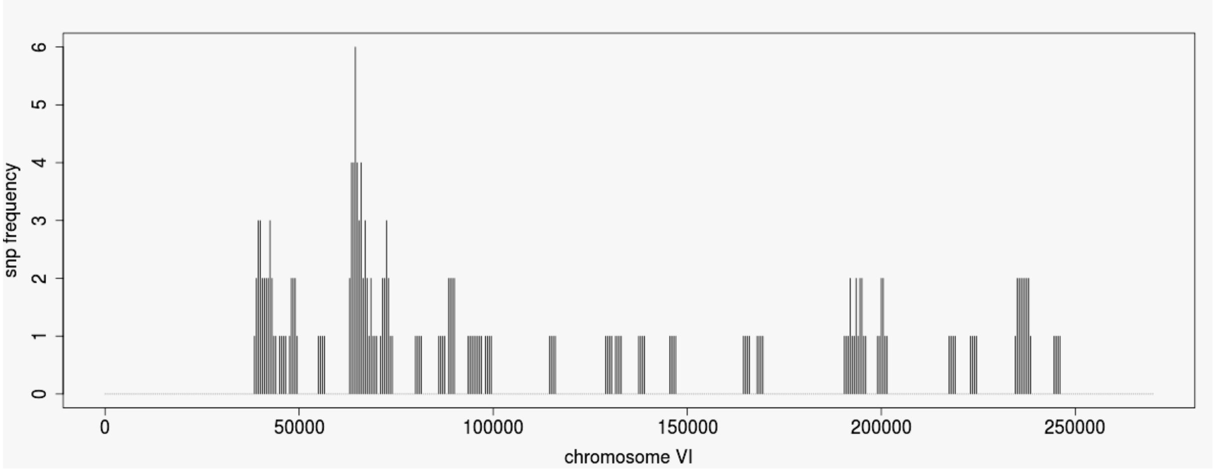
snp frequency
chromosome VI
0
1
2
3
4
5
6
0
50000
100000
150000
200000
250000

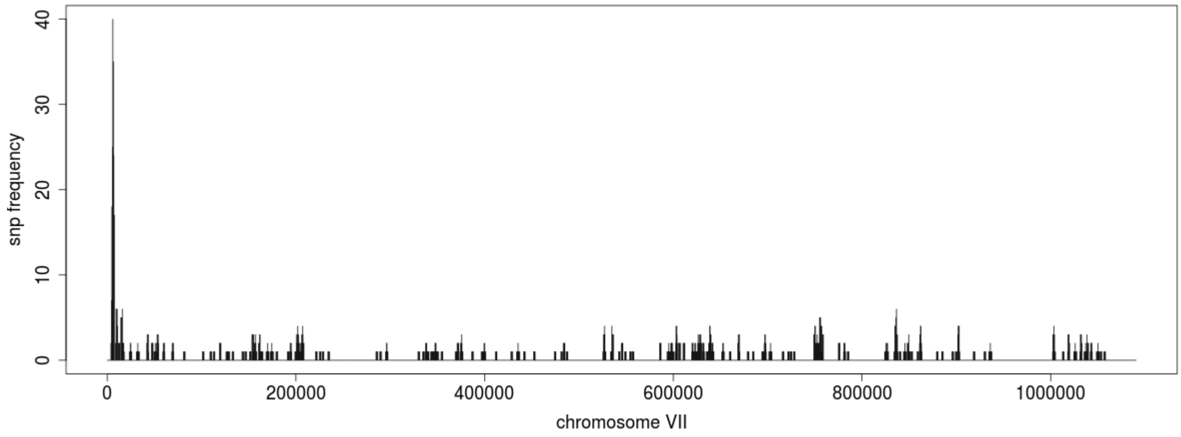
snp frequency
chromosome VII
0
10
20
30
40
0
200000
400000
600000
800000
1000000

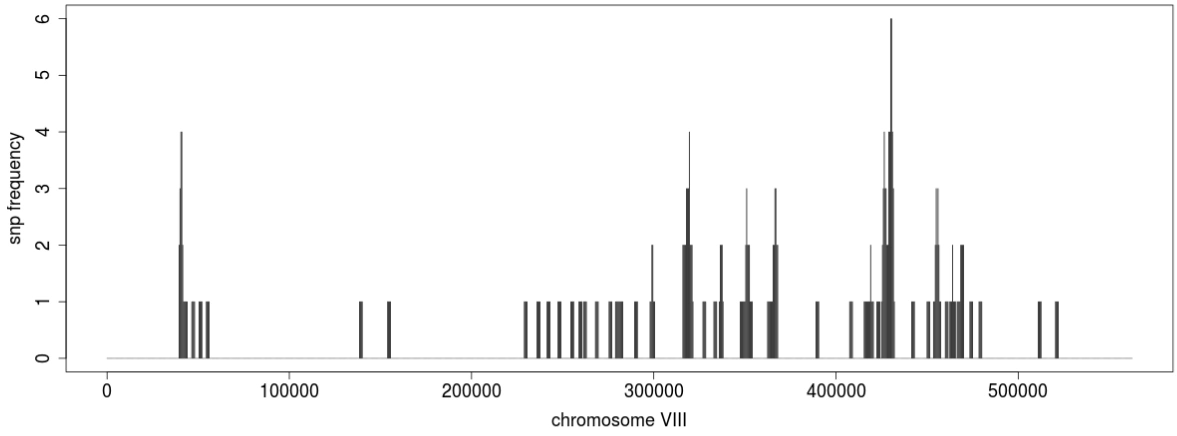
snp frequency
chromosome VIII
0
1
2
3
4
5
6
0
100000
200000
300000
400000
500000

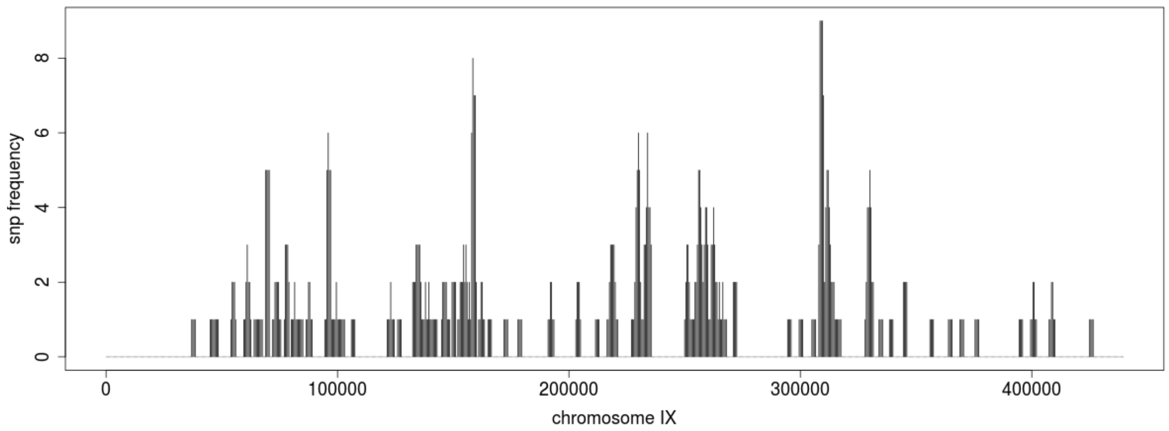

snp frequency
0
2
4
6
8
0
100000
200000
300000
400000
chromosome IX

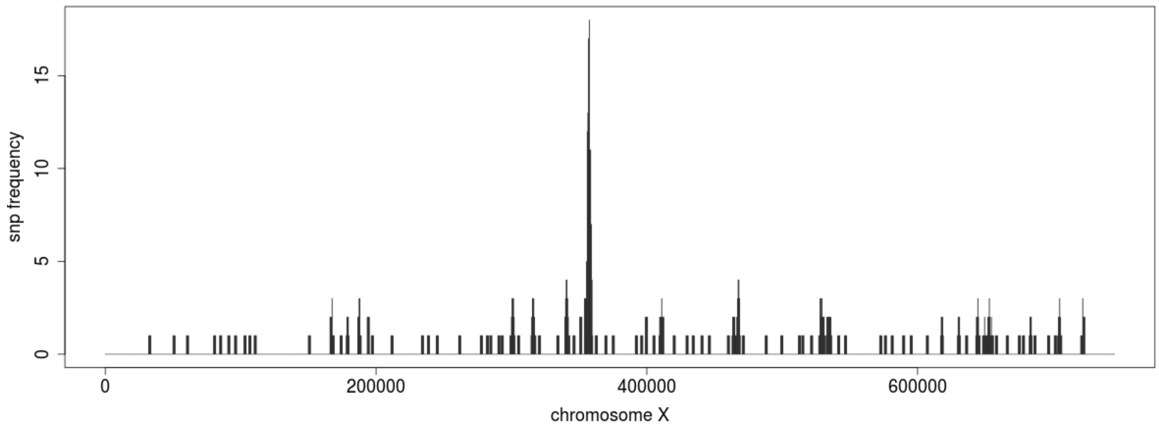

snp frequency
0
5
10
15
0
200000
400000
600000
chromosome X

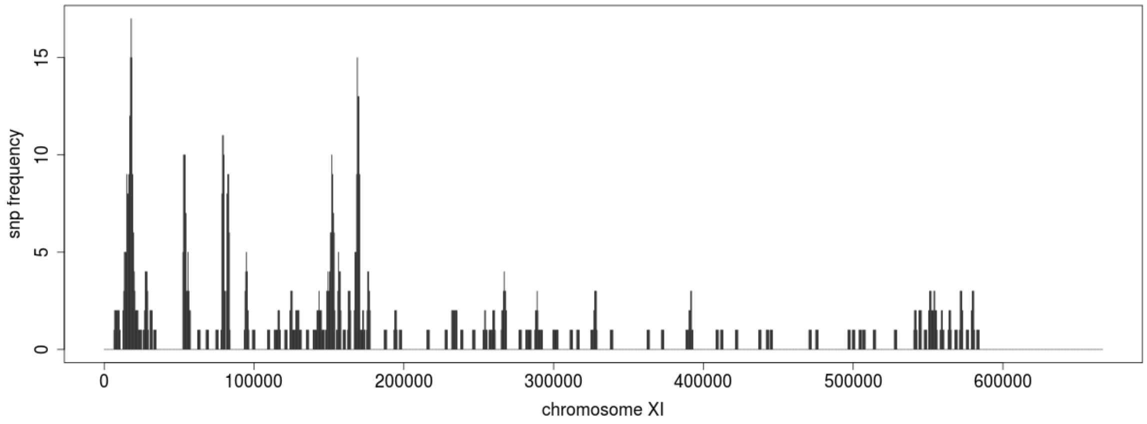

snp frequency
0
5
10
15
0
100000
200000
300000
400000
500000
600000
chromosome XI

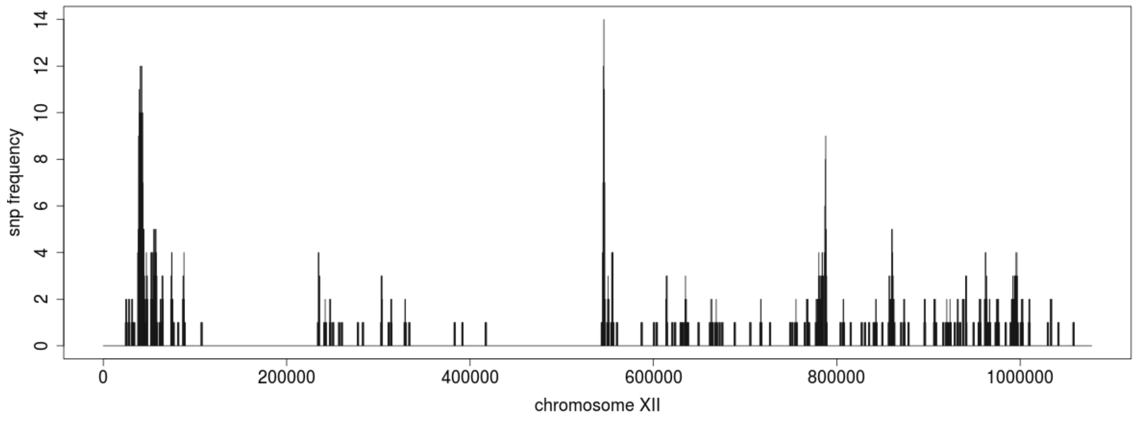

snp frequency
0
2
4
6
8
10
12
14
0
200000
400000
600000
800000
1000000
chromosome XII

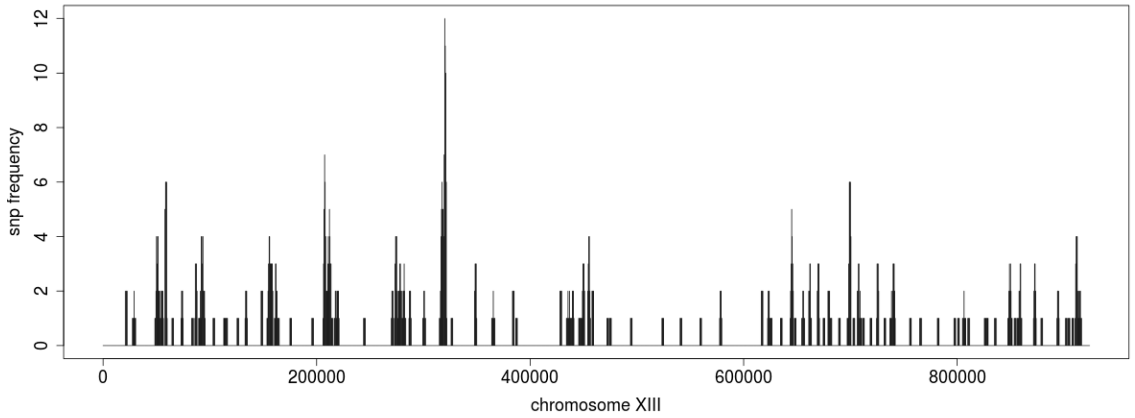
snp frequency
0
2
4
6
8
10
12
0
200000
400000
600000
800000
chromosome XIII

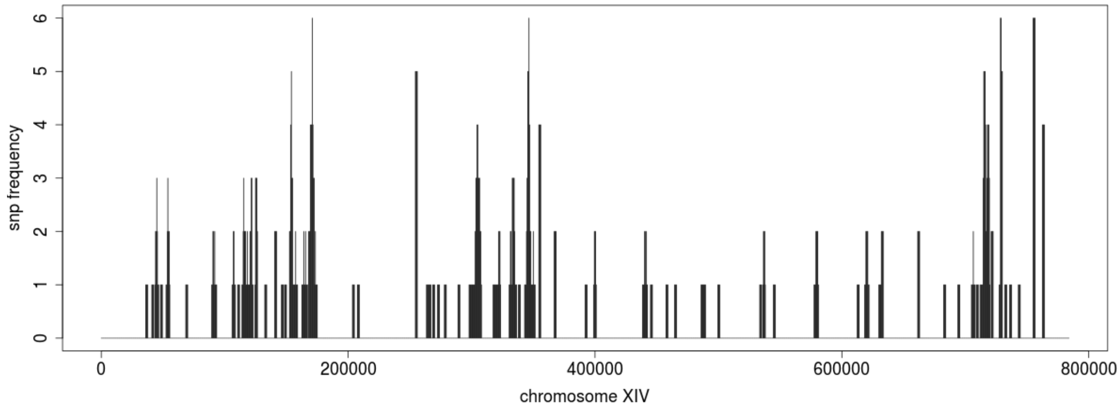
snp frequency
0
1
2
3
4
5
6
0
200000
400000
600000
800000
chromosome XIV

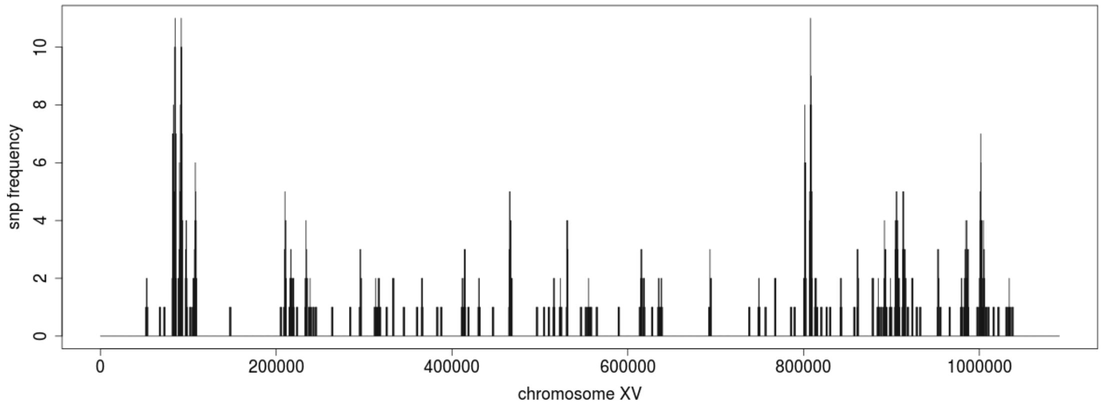
snp frequency
0
2
4
6
8
10
0
200000
400000
600000
800000
1000000
chromosome XV

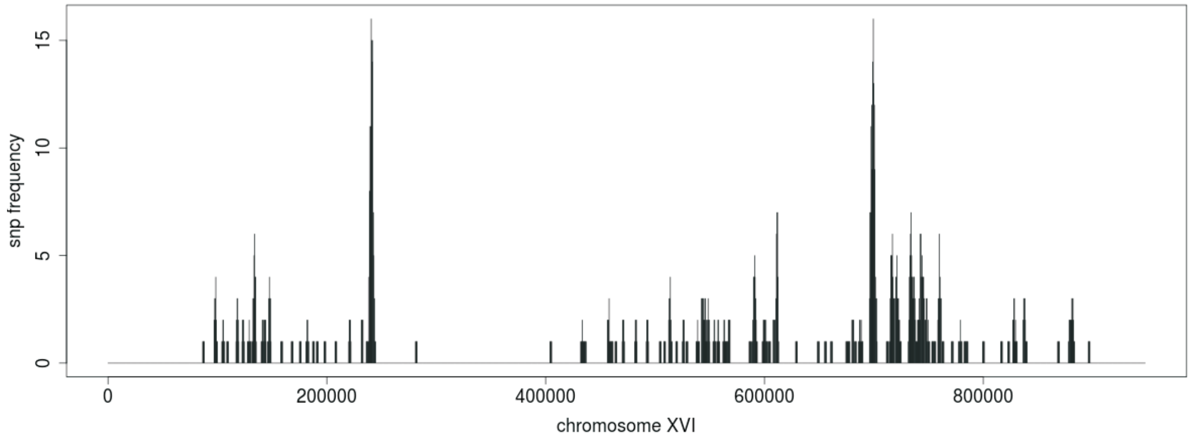
snp frequency
0
5
10
15
0
200000
400000
600000
800000
chromosome XVI
