# Supplementary material for: Whole-Genome Analysis of Three Yeast Strains Used for Production of Sherry-Like Wines Revealed Genetic Traits Specific to Flor Yeasts
Source: Front Microbiol. 2018 May 15;9:965. doi: 10.3389/fmicb.2018.00965 (PMC5962777; doi:10.3389/fmicb.2018.00965)
Supplement: Supplementary file 11 [file Image_2.PDF]

Flo11 (strain I-329)

start-end	sequence
316-340	SSAPV...T...SSTTESSSAPVPTPSSST..T
343-367	SSTPV...T...SSTTESSSAPVPTPSSST..T
370-397	SSAPAptpS...SSTTESSSAPAPTPSSST..T
398-414	...............ESSSAPVPTPSSST..TES
415-439	SSTPV...T...SSTTESSSAPAPTPSSST..T
442-463	SSAPV...T...SSTTESSSAPV...TSST..T
466-490	SSAPV...T...SSTTESSSAPAPTPSSST..T
505-532	SSAPVptpS...SSTTESSSAPAPTPSSST..T
547-571	SSTPV...T...SSTTESSSAPVPTPSSST..T
574-598	SSAPV...S...SSTTESSVAPVPTPSSSS..N
601-628	SSAPS...StpfSSSTESSVAPVPTPSSST..T
629-645	................ESSSAPAPTPSSST..TES
646-670	SSTPV...T...SSTTESSSAPVPTPSSST..T
673-697	SSTPV...T...SSTTESSSAPVPTPSSST..T
700-724	SSTPV...T...SSTTESSVAPVPTPSSST..T
727-751	SSAPV...S...SSTTESSVAPVPTPSSSS..N
769-793	SSTPV...T...SSTTESSSAPVPTPSSST..T
810-839	SSSAPVP.T.PSSSTTESSSAPAPTPSSST..TE
840-869	SSSAPAP.T.PSSSTTESSSAPAPTPSSST..TE
870-885	SSSAPVP.T.PSSSTTES...............
886-910	SSTPV...T...SSTTESSSAPAPTPSSST..T
913-934	SSTPV...T...SSTTESSSAPV...SSST..T
937-961	SSTPV...T...SSTTESSSAPVPTPSSST..T
964-988	SSAPV...S...SSTTESSVAPVPTPSSST..T
991-1015	SSTPV...T...SSTTESSSAPVPTPSSST..T
1018-1042	SSTPV...T...SSTTESSVAPVPTPSSST..T
1045-1069	SSAPV...S...SSTTESSVAPVPTPSSSS..N
1072-1099	SSAPS...StpfSSSTESSVAPVPTPSSST..T
1102-1126	SSTPV...T...SSTTESSSAPVPTPSSST..T
1129-1153	SSTPV...T...SSTTESSVAPVPTPSSST..T
1156-1180	SSAPV...S...SSTTESSVAPVPTPSSSS..N
1187-1210	SSTPF......SSSTESSVAPVPTPSSST..T
1213-1237	SSAPV...S...SSTTESSVAPVPTPSSSS..N

Flo11 (strain S288C)

start-end	sequence
324-339	SSSAPVP.T.PSSSTTES...............
340-364	SSAPV...T...SSTTESSSAPVPTPSSST..T
383-403	....V...T...SSTTESSSAPVPTPSSST..T
442-466	SSAPV...T...SSTTESSSAPVPTPSSST..T
469-493	SSAPV...T...SSTTESSSAPVPTPSSST..T
496-518	SSAPV...T...SSTTESSSAPVPTPSSS....
521-537	................ESSSAPAPTPSSST..TES
538-562	SSAPV...T...SSTTESSSAPVPTPSSST..T
565-587	SSTPV...T...SSTTESSSAPVPTPSSS....
591-620	SSSAPVP.T.PSSSTTESSSAPAPTPSSST..TE
622-645	SSAPV...T...SSTTESSSAPVPTPSSST...
648-677	SSSAPVP.T.PSSSTTESSSAPVPTPSSST..TE
678-701	SSSAPV..T...SSTTESSSAPV.T.SS.T..TE
702-730	SSSAPVP.T.PSSSTTESSSAPVPTPSSST..T
731-743	...............ESSSAPVPTPSSS....
748-771	SSAPV...T...SSTTESSSAPVPTPSSST...
774-802	SSSAPVP.T.PSSSTTESSSAPVPTPSSST..T
803-815	................ESSVAPVPTPSSS....
820-847	SSAPS...StpfSSSTESSSVPVPTPSSST..T
850-876	SSAPV...S...SSTTESSVAPVPTPSSSSniT

Figure S2. Repeat elements in the amino acid sequences of FLO11 proteins in strains I-329 and S288C.

Amino acid sequences of two Flo11 proteins were concatenated and send to the RADAR web server (https://www.ebi.ac.uk/Tools/pfa/radar/help/) to detect repeats. The RADAR output was manually corrected to merge similar repeats and to fix repeats coordinates.

Heger A, Holm L. (2000) Rapid automatic detection and alignment of repeats in protein sequences. Proteins. 41(2): 224-37.
