# Supplementary material for: Whole-Genome Analysis of Three Yeast Strains Used for Production of Sherry-Like Wines Revealed Genetic Traits Specific to Flor Yeasts
Source: Front Microbiol. 2018 May 15;9:965. doi: 10.3389/fmicb.2018.00965 (PMC5962777; doi:10.3389/fmicb.2018.00965)
Supplement: Supplementary file 12 [file Image_3.PDF]

```
Conf:

Pred:

Pred: CCCCCCCCCCCCCCCCCCCCCCCCCCCCCCCCHHHCCCCC
  AA: MEGFNPADIEHASPINSSDSHSSSFVYALPKSASEYVVNH
                      10        20        30        40

Conf:

Pred:

Pred: CCCCCCCCCCCCCCCCCCCCCCCCCCCCCCCCCCCCCCCC
  AA: NEGRASASGNPAAVPSPIMTLNLKSTHSLNIDQHVHTSTS
                      50        60        70        80

Conf:

Pred:

Pred: CCCCCCCCHHHHHCCCCCCCCCCCCCCCCCCCCCHHHHHH
  AA: PTETIGHIHHVEKLNQNNLIHLDPVPNFEDKSDIKPWLQK
                      90       100       110       120

Conf:

Pred:

Pred: HHCCCCCEEEEEECCCCEEEEEECCCCCCCCCCCCCCCCC
  AA: IFYPQGIELVIERSDAFKVVFKCKAAKRGRNARRKRKDKP
                     130       140       150       160

Conf:

Pred:

Pred: CCCCCCCCCCCCCCCCCCCCCCCCCCCCCCCCCCCCCCCC
  AA: KGQDHEDEKSKINDDELEYASPSNATVTNGPQTSPDQTSS
                     170       180       190       200

Conf:

Pred:

Pred: CCCCCCCCCCCCCCCCCEEEECCCCCCCEEEEEEECCCCC
  AA: IKPKKKRCVSRFNNCPFRVRATYSLKRKRWSIVVMDNNHS
                     210       220       230       240

Conf:

Pred:

Pred: CCCCCCCCHHHHHHHHHHHHHCCCHHHHCCCCHHHHHHHC
  AA: HQLKFNPDSEEYKKFKEKLRKDNDVDAIKKFDELEYRTLA
                     250       260       270       280
```

```
Conf:
Pred:
Pred: CCCCCCCCCCCCCCCCCCCCCCEECCCCCCCCCCCCCCCC
  AA: NLPIPTATIPCDCGLTNEIQSFNVVLPTNSNVTSSASSST
                    290       300       310       320

Conf:
Pred:
Pred: CCCCCCCCCCCCCCCCCCCCCCCCCCCCCCCCCCCCCCCC
  AA: VSSISLDSSNASKRPCLPSVNNTGSINTNNVRKPKSQCKN
                    330       340       350       360

Conf:
Pred:
Pred: CCHHCCCCCCCCCCCCCCCCCCCCCCCCCCCCCCCCCCCC
  AA: KDTLLKRTTMQNFLTTKSRLRKTGTPTSSQHSSTAFSGYI
                    370       380       390       400

Conf:
Pred:
Pred: CCCCCCCCCCCCCCCCCCCCCCCCCCCCCCCCCCCCCCCC
  AA: DDPFNLNEILPLPASDFKLNTVTNLNEIDFTNIFTKSPHP
                    410       420       430       440

Conf:
Pred:
Pred: CCCCCCCCCCCCCCCCCCCCCCCCCCCCCCCCCCCCCCCC
  AA: HSGSTHPRQVFDQLDDCSSILFSPLTTNTNNEFEGESDDF
                    450       460       470       480

Conf:
Pred:
Pred: CCCCCCCCCCCCCCCCCCCCCCCCCCCCCCCCCCCHHCCC
  AA: VHSPYLNSEADFSQILSSAPPVHHDPNETHQENQDIIDRF
                    490       500       510       520

Conf:
Pred:
Pred: CCCHHHHHHHHHHHCCCCCCCCCCCCCCCCCCCCCCCCCC
  AA: ANSSQEHNEYILQYLTHSDAANHNNIGVPNNNSHSLNTQH
                    530       540       550       560
```

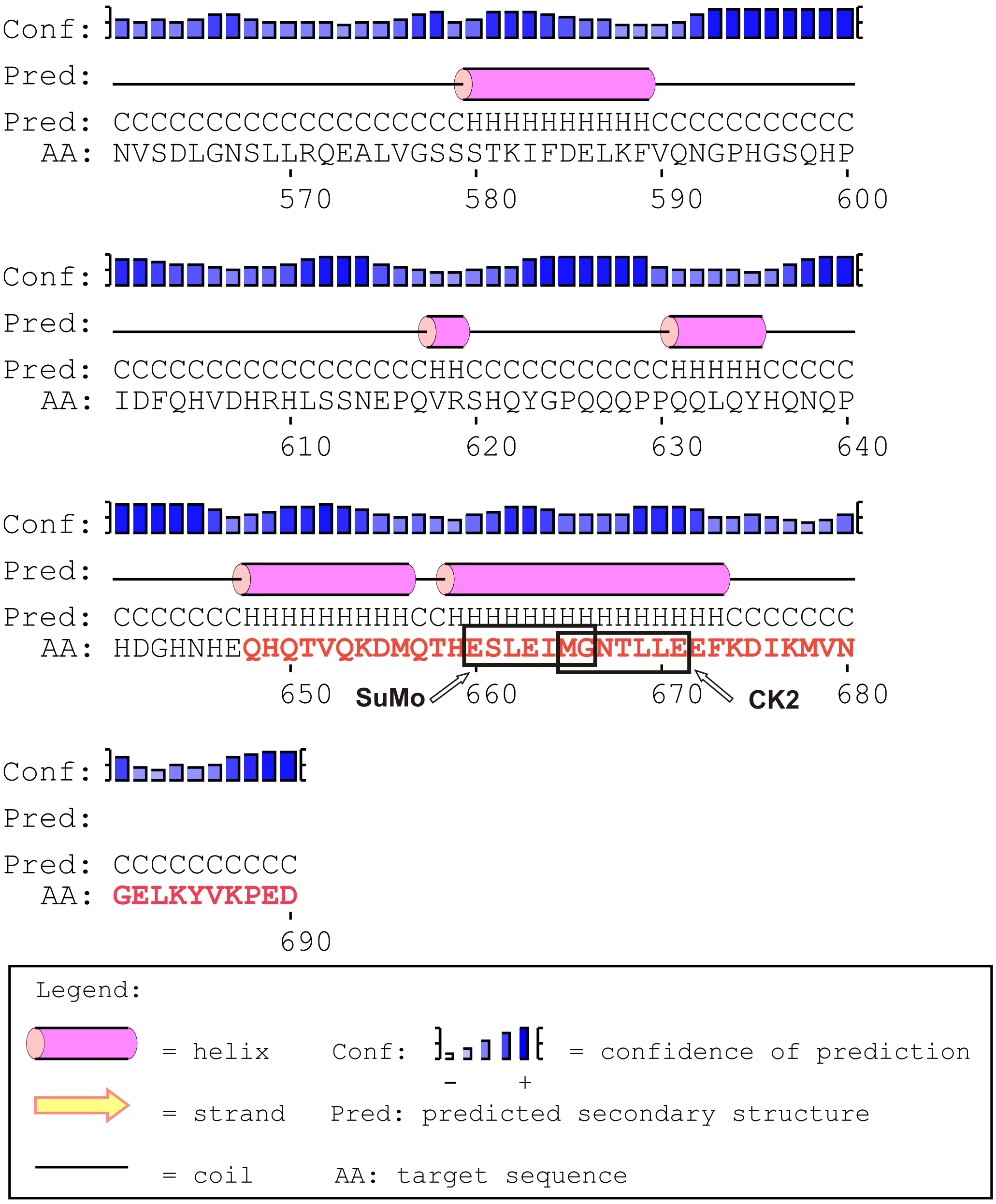

Figure S3. Amino acid sequence of Aft1p and predicted secondary structure. Residues within the C-terminal deletion are highlighted in red. Potential recognition sites for sumoilation and CK2 phosphorylation are boxed (only sites within deletion are shown). Secondary structure was predicted with PSIPRED (Buchan DWA, Minneci F, Nugent TCO, Bryson K, Jones DT. Scalable web services for the PSIPRED Protein Analysis Workbench. Nucleic Acids Research, 2013 41 (W1): W340-W348). Linear motives were predicted using with ELM (Dinkel H, Van Roey K, Michael S, et al. ELM 2016 - data update and new functionality of the eukaryotic linear motif resource. Nucleic Acids Research, 2016, 44: D294-D300)
