# Supplementary material for: Whole-Genome Analysis of Three Yeast Strains Used for Production of Sherry-Like Wines Revealed Genetic Traits Specific to Flor Yeasts
Source: Front Microbiol. 2018 May 15;9:965. doi: 10.3389/fmicb.2018.00965 (PMC5962777; doi:10.3389/fmicb.2018.00965)
Supplement: Supplementary file 13 [file Image_4.PDF]

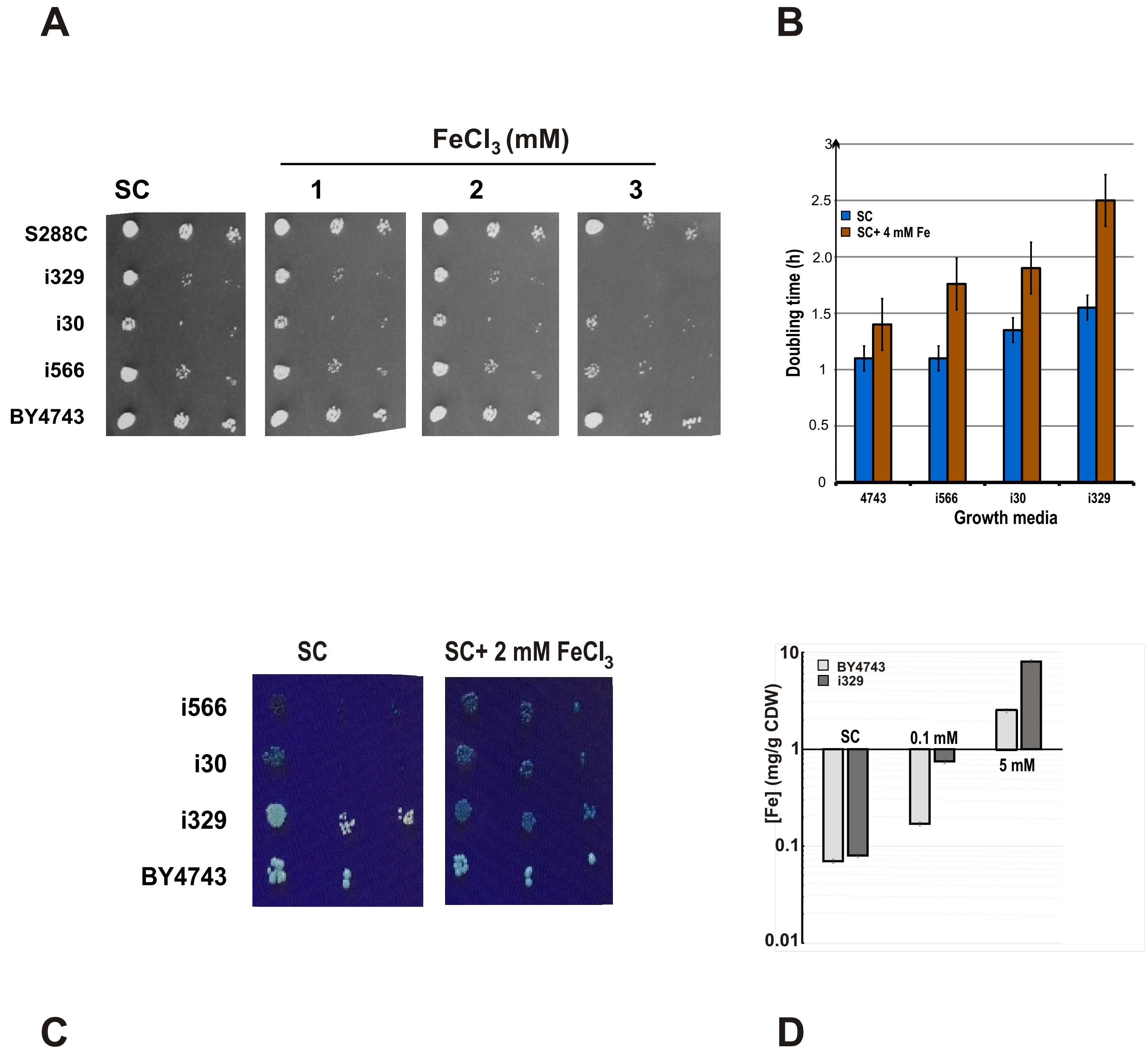

Figure S4. Growth and iron accumulation in selected flor strains under different iron conditions.

(A) Inhibition of flor strains growth on solid media at increased iron concentrations. Three studied flor strains and control BY4743 strain were grown overnight in liquid SC medium and 1:10 dilutions, starting at an OD600 of 0.1, were spotted on solid SC plates with indicated $FeCl_3$ concentrations.

(B) Inhibition of flor strains growth in liquid media at increased iron concentrations. Mean doubling times of flor yeast strains and control strain during log-phase growth in complete synthetic media and in complete synthetic media supplemented with 4 mM $Fe(NH4)_2(SO4)_2$

(C) Iron-dependent methylene blue oxidation by yeast strains. Yeast strains grown overnight in the liquid SC medium and spotted on plates with 1 mM methylene and with and without 2 mM $FeCl_3$ to assess the effect of iron on redox state of yeast cells.

(D) Iron accumulation in BY4743 and i329 strains in media with high and low iron concentrations. Strains were grown in SC medium with varying amount of added $Fe(NH_4)_2(SO_4)_2$. Cells from 5 ml were harvested at OD ~1,0 washed twice with ultrapure water and digested with nitric acid as described (Martínez-Garay, C. A., de Llanos, R., Romero, A. M., Martínez-Pastor, M. T., and Puig, S. (2016). Responses of Saccharomyces cerevisiae strains from different origins to elevated iron concentrations. *Appl. Environ. Microbiol.* 82(6), 1906-1916). Iron levels were determined by inductively coupled plasma-mass spectrometry (ICP-MS) at the “MSU-LAB” Analytical Center of Moscow State University.
